# The complete chloroplast genome of *Mimosa pigra* L. (Fabaceae), a notorious invasive plant

**DOI:** 10.1080/23802359.2022.2086076

**Published:** 2022-06-30

**Authors:** Nguyen Pham Anh Thi, Do Tan Khang, Nguyen Thi Khoa, Van Minh Le, Hoang Dang Khoa Do

**Affiliations:** a Department of Molecular Biotechnology, Biotechnology Research and Development Institute, Can Tho University, Can Tho City, Viet Nam; b NTT Hi-Tech Institute, Nguyen Tat Thanh University, Ho Chi Minh City, Vietnam; c Research Center of Ginseng and Medicinal Materials, National Institute of Medicinal Materials, Ho Chi Minh City, Vietnam

**Keywords:** Caesalpinioideae, chloroplast genomes, giant sensitive tree, Mimosoideae

## Abstract

*Mimosa pigra* L., also called the giant sensitive tree, is native to tropical America and has invaded Africa, Asia, and Australia. Here, we report the complete chloroplast genome of *M. pigra,* which was 165,996 bp in length and composed of a large single-copy region (LSC; 93,299 bp), a small single-copy region (SSC; 17,989 bp) and two inverted repeat regions (IRs; 27,354 bp). The complete *M. pigra* chloroplast genome included 83 protein-coding genes, 37 tRNAs and 8 rRNAs. Phylogenetic analysis using the maximum likelihood method revealed the monophyly of *M. pigra* and related taxa of the subfamily Caesalpinioideae. In comparison, the members of Papilionoideae were paraphyletic.

*Mimosa* L. 1753 is a genus in Fabaceae that includes 612 accepted species (POWO 2021). *Mimosa pigra* L. 1755, also called the giant sensitive plant, is native to America, but has become a notorious invasive plant in various countries in Asia, Africa and Australia (Shanungu 2009; Mansor and Crawley 2011; Rijal and Cochard 2016; Huynh et al. 2020; Witt et al. 2020; POWO 2021). Because it is harmful to agricultural crops, *M. pigra* has been surveyed and controlled in Cambodia, Malaysia, Vietnam, Zambia, and Australia (Shanungu 2009; Mansor and Crawley 2011; Rijal and Cochard 2016; Huynh et al. 2020; Witt et al. 2020). Different phytochemicals have been identified in *M. pigra* extracts (Koodkaew et al. 2018); one extract was shown to protect against cardiovascular diseases (Rakotomalala et al. 2013). Additionally, compounds from a *M. pigra* extract inhibited the growth of other plants, including barnyard grass (Do et al. 2019, 2020). *Mimosa pigra* can also restore polluted soil (Elemike et al. 2019; Pérez-Hernández et al. 2020). These studies revealed both the benefits and disadvantages of *M. pigra*; however, genomic and proteomic researches are required. Therefore, in this study, we sequenced the complete *M. pigra* chloroplast genome using the MiSeq platform to provide the genomic data for future studies of *Mimosa* in particular and Fabaceae in general.

Fresh *M. pigra* leaves were collected in Can Tho, Vietnam (10°02′06.1ʺN 105°46′04.0ʺE) and then stored in liquid nitrogen. No specific permission was required because this species is considered an invasive plant in Vietnam. A specimen was identified by Dr. Nguyen Pham Anh Thi and Dr. Khang Do Tan and deposited at the Biotechnology Research and Development Institute (for free access to the sample, contact Dr. Nguyen Pham Anh Thi; email: npathi@ctu.edu.vn) under voucher number BRDI-THI 20200531-001. Total DNA was isolated using the modified CTAB method (Doyle and Doyle 1987). The DNA extract was used to prepare a sequencing library with a TruSeq Nano DNA Sample Preparation Kit for the Illumina MiSeq platform. The 300 bp paired-end raw reads were imported to Geneious Prime 2021.1 (Kearse et al. 2012) to assemble the chloroplast genome sequence, with *Mimosa pudica* L., 1753 (GenBank acc. no. MH671330) as reference genome. The obtained chloroplast genome was annotated using Geneious Prime 2021.1 and deposited in the NCBI under accession number OL889924.

*Mimosa pigra* had a 165,996 bp in size, typical quadripartite chloroplast genome that includes large single-copy (LSC; 93,299 bp) and small single-copy (SSC; 17,989 bp) regions separated by two inverted repeat regions (IR, 27,354 bp). The genome sequence had a 35.4% GC content and contained 83 protein-coding genes, 37 tRNAs, and 8 rRNAs, of which 16 sequences were duplicated in the IR regions: *rpl2, rpl23, rps12, ndhB, ycf2, trnI-CAU, trnL-CAA, trnV-GAC, trnI-GAU, trnR-ACG, trnN-GUU, trnA-UGC, rrn4.5, rrn5, rrn16,* and *rrn23*. The newly sequenced chloroplast genome showed 83.9% similarity to the chloroplast genome of *M. pudica* in NCBI database (GenBank acc. no. MH671330). The junction between the LSC and IR regions of *M. pigra* was located in the *rps19* coding region (CDS), which is also similar in *M. pudica* cpDNA. Similarly, the junction between the LSC and SSC regions was in the CDS of *ycf1* in both *Mimosa* species. Although the junctions among the LSC/SSC/IR regions are similar in *M. pigra* and *M. pudica*, there are more than 600 species in *Mimosa*. Therefore, more *Mimosa* species should be examined to explore the diversity of junctions between the LSC/SSC/IR regions not only in *Mimosa* but also in Fabaceae.

To conduct a phylogenetic analysis, 78 protein-coding regions in the chloroplast genomes of 18 Fabaceae species were downloaded from the NCBI, of which *Bauhinia blakeana* S.T.Dunn was used as the outgroup. The sequences were then aligned using MUSCLE (Edgar 2004) embedded in Geneious Prime (Kearse et al. 2012) and concatenated. jModeltest 2.0 determined that the best model for the data matrix was the transversion model (TVM) + proportion of invariable sites (I) + gamma distribution (G) model (Darriba et al. 2012). The IQ-TREE package was used to construct a phylogenetic tree using the maximum likelihood method with 1,000 bootstrap replicates (Minh et al. 2020). The phylogenetic tree was illustrated using Figtree (http://tree.bio.ed.ac.uk/software/figtree/). The phylogenetic analysis showed the monophyly of *M. pigra* and related species in the subfamily Caesalpinioideae with high support (Figure 1). In Caesalpinioideae, *M. pigra* and *M. pudica* formed a clade that is sister to the group of *Piptadenia communis* Benth., 1841 and *Stryphnodendron adstringens* (Mart.) Coville, 1910. The complete chloroplast genome provides important information for additional studies on the population genetics of *M. pigra* and possible strategies for controlling its invasiveness.

**Figure 1.**
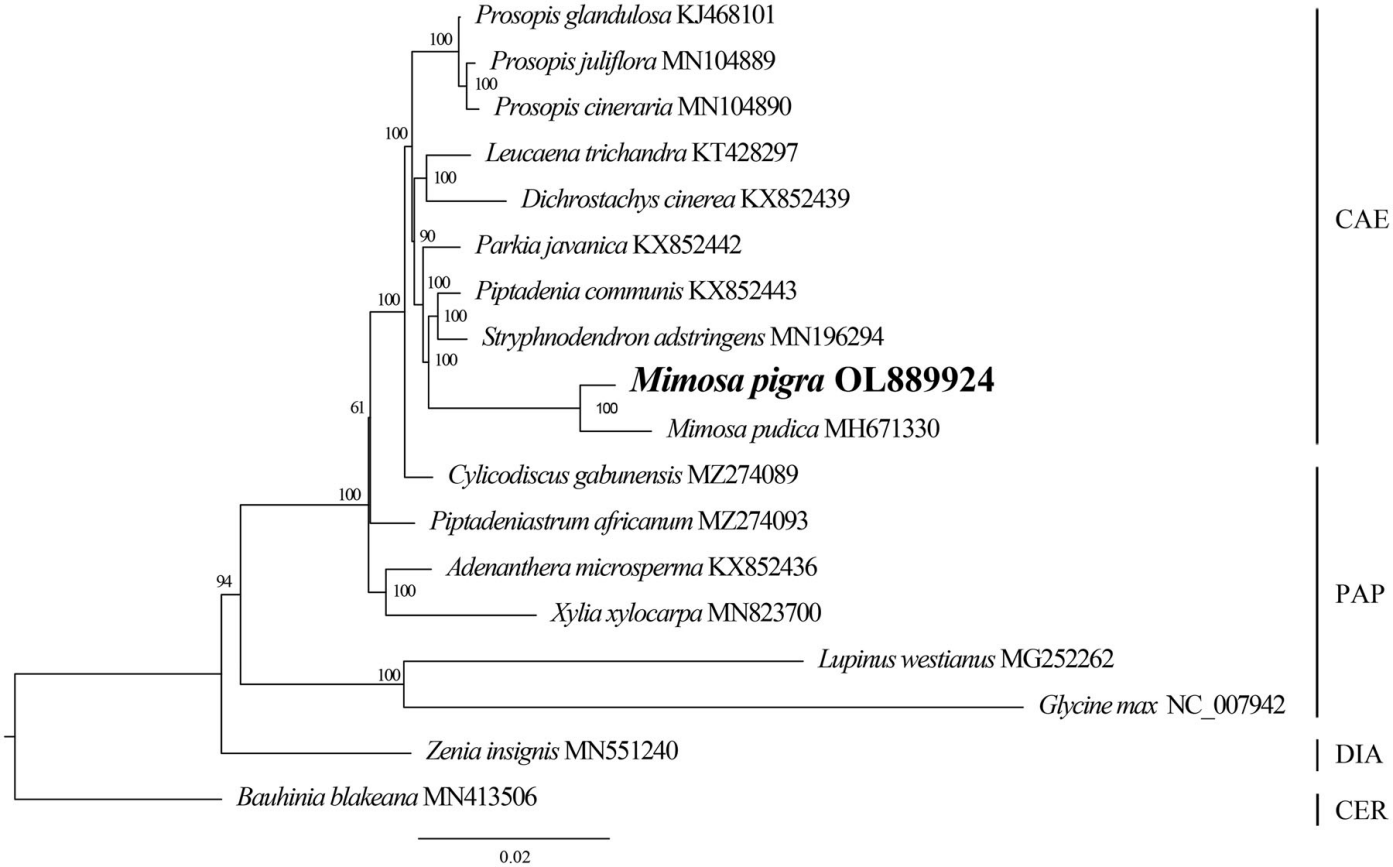
The maximum likelihood tree inferred from 78 protein-coding regions of 18 chloroplast genomes of *Mimosa* and related taxa. The numbers are the bootstrap values. CAE: Caesalpinioideae; PAP: Papilionoideae; DIA: Dialioideae; CER: Cercidoideae.

## Data Availability

The genome sequence data that support the findings of this study are openly available in GenBank of NCBI at https://www.ncbi.nlm.nih.gov] (https://www.ncbi.nlm.nih.gov/) under the accession no. OL889924. The associated BioProject, SRA, and Bio-Sample numbers are PRJNA788179, SRP350381, and SAMN23929642, respectively.
